# Bridging the Human Resource Gap in Primary Health Care Delivery Systems of Developing Countries With mHealth: Narrative Literature Review

**DOI:** 10.2196/mhealth.2688

**Published:** 2013-12-03

**Authors:** Sonu Goel, Nidhi Bhatnagar, Deepak Sharma, Amarjeet Singh

**Affiliations:** ^1^Post Graduate Institute of Medical Education and ResearchSchool of Public HealthChandigarhIndia

**Keywords:** mHealth, human resource, health, developing countries, projects

## Abstract

**Background:**

Mobile health (mHealth) has the potential to solve human resource issues in the health care sector. mHealth is of particular interest in developing countries, where widespread mobile networks and access to devices are connecting people like never before.

**Objective:**

The aim of this paper was to review published and unpublished literature, field projects, and pilot studies on mHealth usage in overcoming shortage of human health resources in developing countries.

**Methods:**

A narrative literature review was undertaken using an iterative approach in extracting literature focused on mHealth and human health resources of low-income countries, especially India. The present review has undertaken comprehensive coverage of the work on related field projects that have been either published, accepted for publication, or pilot tested.

**Results:**

This review presented the use of mHealth across various dimensions of primary health care, including data collection, disease surveillance, health education, supervision, monitoring, and feedback. Field studies of fast, error-free data collection and transmission using mHealth were also documented. New apps for supervision, monitoring, and utilization of innovative health education tools were documented in the current review. Practical limitations of mHealth and challenges set forth in developing countries included issues of data security, cost constraints, health provider privacy, and technical barriers.

**Conclusions:**

In the present review, we have documented a few mHealth projects that contribute to the proficient use of human resources. These projects pave the path for the efficient utilization of mHealth, offering solutions to emerging human resource challenges and simultaneously revamping the health care delivery in resource-limited settings.

## Introduction

The world today is experiencing an extraordinary progress in the field of interpersonal communication. Several developing countries such as India have witnessed an explosive annual growth rate of nearly 39% in wireless subscriber base during the year 2010-2011 [1]. Mobile technologies have already changed and will continue to make an impact on the lives of millions. Mobile phones, because of their high penetration levels, hold tremendous potential and provide opportunities never imagined before in the areas of communication, entertainment, education, employment, and health. However, the health sector has been slower in adopting mobile technologies into routine operations, and its adoption would benefit patients and providers alike.

The health sector faces several challenges, and many of these problems could be circumvented by the intelligent use of mobile phone technology. Inadequacy of human resources in the health sector is an identified bottleneck in achieving universal access to quality health care [2,3]. Health workforce is defined as “all people engaged in actions with primary intent to enhance health” [4]. According to the World Health Report 2006, a shortfall of health workforce has been documented in 57 countries. The scenario is worse in Southeast Asian regions, which accommodate 25% of the world’s population and take 30% of the global disease burden, as these have only 10% of the global health workforce [5]. In India, 20 health workers and 1 doctor for every 10,000 people translates to a shortage of nearly 2.6 million health workers [6]. The health workforce inequity exists between genders, regions, and categories of health workers [7]. At present, mobile phones with their reach, connectivity, and features of smartphone have the capability to provide potential solutions to many of these challenges.

Mobile health (mHealth) is a relatively recent term for medical and public health practices supported by mobile devices, such as mobile phones, patient monitoring devices, personal digital assistants (PDAs), and other wireless devices. The term “mHealth” was coined by Professor Robert Istepanian as the use of “emerging mobile communications and network technologies for healthcare” [8]. It is a simple, low-cost, and immensely user-friendly service capable of enhancing the speed and accuracy of health care delivery. mHealth applications include the use of mobile devices for collecting community and clinical health data; delivery of health care information to practitioners, researchers, and patients; real-time monitoring of patients’ vital signs; and direct provision of care (via mobile telemedicine) [9].

Interest in the application of mobile phone technology has been growing in developing countries, where widespread mobile networks and access to devices are connecting people like never before. The present paper attempts to present a literature review on the utilization of mHealth services for bridging human resource gap in the health care sector. It is expected that the summary of the extent of utilization of this technology in current field situations presented in this review will guide any future work in this domain.

## Methods

The present paper presents a narrative literature review using an iterative approach in extracting relevant literature from developing nations on the utilization of mobile technology for bridging human resource gap in health care sector. A comprehensive coverage of work on many papers and field projects that had either been published, unpublished, or are being pilot tested has been ensured. The search was restricted to literature sources in English; however, no time restriction was imposed. Search engines such as PubMed, IndMed, Directory of Open Access Journals, and Google Scholar were used to identify published and unpublished/in-progress studies, references, and citations of articles of interest. Search terms that were used in the process were “mHealth,” “projects,” “health manpower,” “human resource,” “rural,” “underserved area,” and “India.” These terms were searched individually and in conjunction with professional classifications such as health workers, nurses, doctors, and midlevel workers. All studies, project reports, and policy statements that dealt with the use of mobile phones for epidemiological purposes in health, for example, data collection, supervision and feedback, health promotion, and workload reduction, were included in the study. In addition, manual search of literature was also performed in the institute library to collect relevant information. A systematic search of pertinent journals, such as *Journal of Medical Internet Research* and *Indian Journal of Medical Informatics*, was also undertaken*.* Unpublished literature (using authors’ knowledge of the field) was included in the review along with inputs from experts using the snowballing technique to identify additional studies. The websites with mHealth project databases, for example, Royal Tropical Institute mHealth project database for low-resource settings, were searched for key projects on mHealth. Reports of using audiovisual aids, computers, and e-devices in hospitals and health care sector were excluded from the review. mHealth projects that appeared to have no influence on the work burden or bridging manpower gap were also excluded from the study. Finally, a total of 28 relevant projects on mHealth were included in the review.

## Results

### Primary Results

mHealth projects have been initiated on various dimensions of health care, for example, data collection, surveillance, training, health education, awareness, supervision, and monitoring. A large-scale implementation of such projects could become the forerunner of health care reforms in the future. mHealth applications and tools can significantly contribute to reducing the workload and improving the performance of health care workers. In addition, skill mix of health care workers is ensured as they perform multiple tasks through smartphone aids. Thus, this results in the twin benefits of reducing human resources needed for various tasks and greatly improving service quality.

### Data Collection and Disease Surveillance

Paper-based field data collection and transmission has remained a cost-intensive and time-consuming activity until now. But using mobile applications helps in transmission of real-time data, quick calculations, easy transfers, and distant supervision. Mobile applications help in lowering cost, saving time, and improving data accuracy by reducing the possibility for human error and avoiding duplication of reports. Health surveys, in context of mHealth, are defined as the use of mobile devices for health-related data collection and reporting.

The pilot project led by International Centre for Diarrheal Disease Research and National Institute of Preventive and Social Medicine was conducted in Bangladesh using PDAs to collect data [10]. PDAs were also successfully deployed to collect data for Global Adult Tobacco Survey in India [11].

Smartphones enable health care workers to download several health-related applications. EpiSurveyor, a mobile data collection tool, offers data collection forms for medical projects. In 2008, Kenyan health workers were successful in preventing a potential polio epidemic using online software to track emergency vaccination campaign against poliomyelitis. In 2010, the same mobile phone application was field tested in Malawi for feasibility and scalability in a pilot project to monitor availability of malaria medicines. The project helped provide quality services and reduce the workload of health workers [12]. Curioso et al, in their study in Peru, observed that self-designed Cell-PREVEN system had proven effective in real-time reporting of adverse events from health workers, or from a doctor reporting outbreak of a disease [13]. Similarly, Media Lab Asia, in collaboration with All India Institute of Medical Sciences, New Delhi, India, helped in creating accessible and sustainable health system to the community in which an open source software application was used to collect medical and demographic data. In this effort, a high adoption of the technology was observed and also a significant reduction in total time needed for data entry was noted [14].

A door-to-door cancer campaign was conducted in the state of Punjab, India, in 2012 covering 2.3 million people. Health workers used simple mobile phones for data transfer, and negligible errors were reported in the process. This process helped in completing the survey in a record time of 20 days, as use of mobile phones significantly reduced transport time and separate time required for data entry and transfer [15].

Mobile phones can be appropriately utilized as a cost-effective tool for real-time disease surveillance. Quick dissemination of health information can enable health administrators with limited field staff and supervisors to take timely action. mHealth helps in real-time monitoring of diseases and public health problems, thereby reducing the workload on health workers.

Muthiah et al investigated the effectiveness and efficiency arising out of the application of mobile phone technology for early detection of disease outbreaks on a real-time basis from July 2008 to July 2010. The data were collected from outpatient health units of primary health centers and subcenters using mobile applications. Adverse events were rapidly disseminated via text messaging (short message service, SMS), email, and through Internet using Web-based software. The entire process of data collection and reporting was highly efficient [16].

The Community Health Information Tracking System (CHITS) in Philippines empowered local communities with information and enabled a 2-way data flow using mobile technology. CHITS trained local health workers on how to use the information system, enabling them to take action and empower others [17]. A pilot project Real-Time Biosurveillance Program was implemented by Sarvodaya and LIRNEasia to investigate the conditions for effective deployment of wireless technologies in disease surveillance. This project proved effective in the early detection of clusters of population affected by chicken pox, acute diarrheal disease, respiratory tract infection, dengue, and viral fever in Kurunegala district, Tamil Nadu, India [18].

Mobile phones have been pilot tested extensively in the communicable disease surveillance programs of various developing nations: for example, malaria in Uganda and Botswana, dengue in Mexico, tuberculosis (TB) in Pakistan, infectious disease surveillance after earthquake in China, and setting up an electronic disease surveillance and response system in Tanzania [19].

Mobile phones were found acceptable and feasible in the collection of maternal and child health data from women living with human immunodeficiency virus (HIV) in South Africa [20].

To summarize, mHealth technology has proven effective in increasing the efficiency of data collection and disease surveillance with reduced human resource requirements.

### Health Education and Training Tool

Health information and messages can be disseminated on a large scale to public using mobile phones. Commercial films and videos delivered on the mobile phones can help in improving the efficiency of health education sessions in outreach sessions where elaborate audiovisual aids cannot be arranged.

AIDS was a taboo in Georgia but the adoption of mobile phones was high. Mobile phones were effectively used for disseminating knowledge about HIV/AIDS through video films featuring popular actors. This fast transfer of knowledge on HIV through video was the first kind of use of mHealth for education and awareness in Georgia. This paved way for other successful programs utilizing mHealth in the country [21].

Mobile phone games have also been used in the fight against the spread of HIV/AIDS. The project, Freedom HIV/AIDS in Africa, was the first-ever initiative to focus on HIV/AIDS awareness using mobile phone games. By Play-and-Learn method, these games make learning exciting and help in the enhancement and retention of knowledge among children [22].

Project Masiluleke in South Africa focused on raising HIV awareness. It encouraged people to get tested for HIV/AIDS and to undergo treatment by ensuring that those who tested positive adhered to individualized treatment regimens. In the first phase of project (2009), text messages were sent to encourage utilization of testing and treatment services for HIV/AIDS. This significantly increased call volume to National AIDS Helpline with a large number of people reaching out to health workers for information [23].

African Medical and Research Foundation, an international African organization, initiated a pilot project through which community members in 4 districts received TB-related quiz questions and awareness messages in local dialect as well as in English through mobile phones. Participants who successfully completed quiz questions were rewarded with prizes (eg, free airtime, mobile phones, and T-shirts). This contributed to significant increase in the number of TB patients taking medication [24].

A nongovernment organization in Uganda, Text to Change, created awareness about HIV/AIDS by providing an SMS-based quiz to a large number of mobile phone subscribers. It focused on improving health education through text messaging, using anonymity and capability of mobile having a greater reach among population. This program aimed to encourage citizens to seek voluntary testing and counseling for HIV/AIDS. Free airtime was offered to users to encourage their participation in the program. The quiz was interactive and focused on general knowledge about HIV transmission and the benefits of voluntary testing and counseling [25]. Similarly, AIDS hotline and free AIDS text messages in Ethiopia, HIV Confidant project in South Africa, and Jalaaka Project in India facilitated education and spreading awareness about HIV among the community and high-risk groups [19].

mHealth has also been used for educating community in projects focused on family planning, adolescent health, and antenatal care. CycleTel, a mobile-based application launched in India, helps the user implement family planning using Standard Days Method. It calculates fertile period for women and advocates sex avoidance during that period [26]. Community health workers in Bihar, India, used mobile phone application CommCare to disseminate information on common adolescent health issues such as menstrual hygiene, sexually transmitted diseases, and family planning methods among adolescent girls and women. The outcome of the project was encouraging in that it had a wider outreach among adolescents, even as health workforce was scarce [27].

Projects that used mobile phones to access the community to provide them information and education, as well as for communication, were developed by several nonprofit organizations. Project mDHIL Health Information on mobiles in India, Project Zumbido for HIV/AIDS in Mexico, and Sex-Ed-Thru Text in Indonesia are some examples of such projects [28].

Thus, behavior change communication through mobile phone eliminates the need for a larger health workforce, as it has the capability to reach a large section of the population automatically and creates considerable impact.

### Supervision and Monitoring

Supervision in health care system is suffering due to lack of role clarity, time, and cost constraints, in addition to poor access to peripheral health centers. Supervisors, at all levels, are overburdened with their primary responsibilities. Therefore, a supervisor’s workload is reduced through a mobile application, using which long-distance supervision can be performed and feedback delivered through mobile apps. It reduces the frequent need for on-site supervision without compromising work quality.

Tracking of health workers using global positioning system (GPS) technology or viewing location of employees on Google Maps installed on their mobile phones has proven very effective in streamlining management oversight and improving worker attendance. Econz Timecard GPS is one such mobile time card application enabling supervision through mobile technology [29].

A project on malaria monitoring, diagnosing, and treatment was launched in Botswana in 2012 with the assistance of local mobile network providers. Local health workers were trained to diagnose malaria by conducting rapid diagnostic tests. The results were reported via mobile phones, offering real-time access to malaria data, trends, and locations. The information collected proved useful for decision makers in planning vital elements of malaria control program, for example, supply of adequate malaria medicines, bed nets, and sprays [30].

Alam et al (2010), in their study on assessing the scope of mobile-based solutions to improve maternal and child health in Bangladesh, showed that by installing a smart algorithm in mobile phones of health workers, delivery of efficient health services to pregnant mothers and neonates was possible with reduced human resources [31].

Mother and Child Tracking System under National Rural Health Mission in India was introduced to ensure delivery of comprehensive maternal and child health services through the use of mobile phones. Work plans and reminders for antenatal and postnatal checkups were sent via mobile phones to respective health workers. This eliminated the need for paper work to draft the work plan and manual delivery. Repeated reminders through phone calls and SMS ensured better and timely health care delivery to the beneficiaries [32].

Similarly, female health workers in Pakistan were provided with mobile phones to remain in touch with the officials and provide efficient service delivery to the community [33]. Rapid SMS project in Rwanda in Africa enabled community health workers to track pregnant women, monitor antenatal care, identify and refer women at risk, and improve communication with health facilities during emergencies [34].

A Web-based application, Colecta-PALM delivered on PDAs, was used for sending messages about behavior based on risk-assessment responses to people living with HIV and AIDS in Peru [35]. Similarly, SMS printers linked to HIV testing centers were successfully pilot tested in Mozambique. The test results, stored in the health centers, were immediately available through a single command from the HIV center [36].

Project Mobile-DOTS for tuberculosis patients in Kenya (2008) had been successful in drug delivery to TB patients. Treatment supporters were asked to capture videos of patients as they took medications daily and view motivational and educational text and video health messages. This was submitted for review by health professionals. It was observed that most patients preferred MDOT to clinic DOT (patient visiting health center for TB drug administration) or DOT delivered by visiting community health workers [37].

Although not rigorously tested, regular monthly review of remote health facility functions can be performed effectively via mHealth applications. This eliminates the need for physical presence of health workers for the monthly meeting and frees their time to focus on their activities for implementing national health programs.

A vast majority of people in rural areas make decisions on where to seek treatment primarily based on the consultations they make with the field worker. Instead, health workers can deliver information to these people about the available health facilities through text messages. A similar SMS-based network was established in Cambodia to gather data on adverse events following immunization and guide patients for immediate referral as soon as a danger sign was noticed. Mobile phones can thereby help in setting up referral mechanisms and maintaining smooth patient flow in the process [38].

A pilot project was undertaken by the School of Public Health, Postgraduate Institute of Medical Education and Research, Chandigarh, in the area of rural field practice in 2006 with the objective of enhancing the outreach of health care services. A senior resident (public health specialist) gave his mobile phone number stamped on outpatient department slips to patients, municipal committee members, anganwadi workers, sarpanches, and mahila mandals for a period of 3 months. The phone calls that the senior attendant received were answered, a record of which was duly maintained in the personal pocket diary of the senior resident. Callers sought information on availability of doctor, health problems, and immunization services. The study concluded that public at large is favorable to the idea of mobile phone usage for medical consultation [39].

### Feedback Mechanism

mHealth can be utilized in feedback assessment of health services. An interactive voice response system allows the user to provide feedback on health care service delivery. Supervisors can send messages to people asking for feedback and for tracking service delivery in their area of work using mHealth. Business organizations and mobile operators have mHealth systems in place already. Data generated through the feedback process can be used to rank health care facilities, thereby contributing to improving the health care system and performance of people. In many countries, helpline phone numbers exist for patients to call for medical consultations and help. In India, health helpline (phone number 104) is provided by Health Management and Research Institute in collaboration with state governments (Maharashtra, Rajasthan, Assam, and Andhra Pradesh). At a very minimal cost, any citizen can have medical consultation or lodge a service complaint against any public health facility.

## Discussion

### Challenges in mHealth

Most of the mHealth projects are funded by external donors and are not self-sustaining, thereby affecting long-term sustainability of such initiatives. Few shortcomings such as loss of privacy, cost issues for sustainability, nonavailability of responsive technology support, and lack of training support were noticed in using mHealth for data collection and reporting. Attitudinal barriers of health care providers toward mHealth must be addressed effectively. Some issues with regard to technical feasibility in the form of doubts about the quality of data entry and analysis also arise. Data security is another important issue in using mHealth applications. Using mHealth technologies causes various legitimate concerns about the security of citizen information collected for various programs. In particular, security lapse in message transmission and data storage can lead to compromising the collected data if the necessary precautions are not taken. Health hazards of radiation emitted from mobile phones is another concern drawing attention. Moreover, initiating mHealth project is a cost-intensive activity. A prerequisite to provide mobile phones to the workers or reimburse their phone bills should be inbuilt into the program. Otherwise, it adds to the financial burden on the worker, leading to sustenance issues. Privacy concerns of health workers in mHealth must be clearly addressed as it has been observed that many irrelevant calls are made at odd hours.

### Conclusions

To conclude, mHealth care can bring about a *revolution* in health care in India and other developing nations. Vast reach and penetration of this technology can be effectively harnessed for health care delivery where access is the key barrier. Investment in mHealth reduces spending on stationery, travel, and time, and thus meets the challenge of shortage in human resources (both quantity and quality). Moreover, adoption of mHealth by workers in their day-to-day work helps them improve their productivity and efficiency, which is translated into enhancing quality and job satisfaction. Continuing health education of workers through mobile phones helps them remain motivated and up-to-date with the latest guidelines and developments.

mHealth is a new development in health care, the potential of which remains underutilized. A need to go beyond the pilot testing of projects and scale it to the national level is required [40]. Well-designed research studies are needed to explore existing challenges in mHealth and experiment with newer applications. Issues such as large capital cost, data security, disturbed privacy, and sustainability of several projects require great attention. The present review provides an overview of some key mHealth projects and studies undertaken in developing countries. Considering human resources crunch and issue of poor accessibility in rural and underserved areas of India, mHealth is the only existing viable solution.

## Abbreviations

CHITS: Community Health Information Tracking System
GPS: global positioning system
HIV: human immunodeficiency virus
mHealth: mobile health
PDA: personal digital assistant
SMS: short message service
TB: tuberculosis
